# Self-Reported Lifetime History of Eating Disorders and Mortality in the General Population: A Canadian Population Survey with Record Linkage

**DOI:** 10.3390/nu13103333

**Published:** 2021-09-23

**Authors:** Pardis Pedram, Scott B. Patten, Andrew G. M. Bulloch, Jeanne V. A. Williams, Gina Dimitropoulos

**Affiliations:** 1 Department of Psychiatry, University of Calgary, 2500 University Drive NW, Calgary, AB T2N 1N4, Canada; gdimit@ucalgary.ca; 2 Mathison Centre for Mental Health Research & Education, Foothills Medical Centre, 3280 Hospital Drive NW, Calgary, AB T2N 4Z6, Canada; patten@ucalgary.ca (S.B.P.); bulloch@ucalgary.ca (A.G.M.B.); 3 Hotchkiss Brain Institute, University of Calgary Foothills, 3330 Hospital Drive NW, Calgary, AB T2N 4N1, Canada; 4 Department of Community Health Sciences, University of Calgary, 2500 University Drive NW, Calgary, AB T2N 1N4, Canada; jvawilli@ucalgary.ca; 5 O’Brien Institute for Public Health, University of Calgary Foothills, 3280 Hospital Drive NW, Calgary, AB T2N 4Z6, Canada; 6 Cuthbertson & Fischer Chair in Pediatric Mental Health, 2500 University Drive NW, Calgary, AB T2N 1N4, Canada; 7 Faculty of Social Work, University of Calgary, 2500 University Drive NW, Calgary, AB T2N 1N4, Canada

**Keywords:** eating disorder, all-cause mortality, epidemiology, hazard ratio, general population, Canada

## Abstract

Eating disorders (EDs) are often reported to have the highest mortality of any mental health disorder. However, this assertion is based on clinical samples, which may provide an inaccurate view of the actual risks in the population. Hence, in the current retrospective cohort study, mortality of self-reported lifetime history of EDs in the general population was explored. The data source was the Canadian Community Health Survey: Mental Health and Well-Being (CCHS 1.2), linked to a national mortality database. The survey sample was representative of the Canadian household population (mean age = 43.95 years, 50.9% female). The survey inquired about the history of professionally diagnosed chronic conditions, including EDs. Subsequently, the survey dataset was linked to the national mortality dataset (for the date of death) up to 2017. Cox proportional hazards models were used to explore the effect of EDs on mortality. The unadjusted-hazard ratio (HR) for the lifetime history of an ED was 1.35 (95% CI 0.70–2.58). However, the age/sex-adjusted HR increased to 4.5 (95% CI 2.33–8.84), which was over two times higher than age/sex-adjusted HRs for other mental disorders (schizophrenia/psychosis, mood-disorders, and post-traumatic stress disorder). In conclusion, all-cause mortality of self-reported lifetime history of EDs in the household population was markedly elevated and considerably higher than that of other self-reported disorders. This finding replicates prior findings in a population-representative sample and provides a definitive quantification of increased risk of mortality in EDs, which was previously lacking. Furthermore, it highlights the seriousness of EDs and an urgent need for strategies that may help to improve long-term outcomes.

## 1. Introduction

Eating disorders (EDs) are serious and persistent psychiatric disorders characterized by severe disturbance in body weight and eating behavior [1]. Based on the Diagnostic and Statistical Manual of Mental Disorders-Fifth Edition (DSM-5) classification, EDs include anorexia nervosa, bulimia nervosa, binge eating disorder, other specified feeding or eating disorders, and avoidant/restrictive food intake disorder [2]. Based on the systematic review of 33 studies, the lifetime prevalence of EDs (total) is 8.4% for women and 2.2% for men [1], that of anorexia nervosa is 1.4% for women and 0.2% for men, that of bulimia nervosa is 1.9% for women and 0.6% for men, and finally, that of binge eating disorder is 2.8% for women and 1.0% for men [1]. EDs are associated with significantly impaired health-related quality of life compared with the healthy population and even with those with other psychiatric conditions [3]. These disorders encompass a range of problematic behaviors, including starvation, binge eating, and purging, leading to an increased risk of premature death [4]. Many factors in these patients have also been identified as predictors of mortality, such as type of ED diagnosis, low body mass index (BMI), suicide behaviors, alcohol abuse, and comorbidities [5,6,7]. In addition, the age of onset and age of treatment are also two important death-predictive factors in patients with EDs, as evidence has shown that older individuals (25–44 age group) have an elevated risk of mortality for all types of EDs compared to youth (15–24 age group) [8]. Mortality data on EDs are important and considered an indicator of illness severity [9]. Most mortality studies in EDs have focused on anorexia nervosa; however, a few studies of bulimia nervosa and other specified feeding or eating disorders, and even fewer for binge eating disorder, have been published [4]. The standard measures for mortality are the crude mortality rate (CMR) (CMR is the proportion of death within the study population over a specific period) [10], the standardized mortality rate (SMR) (SMR is calculated using the number of observed deaths in a targeted population at a certain point of time divided by the number of expected deaths in the general population while taking into account certain demographic variables) [11], and hazard ratios (HR) (the HR in survivorship curves is the temporal progression of death within a group and defined as the hazard in the groups with EDs divided by the hazard in the control groups) [12,13]. 

The ED mortality studies are largely based on cohorts from inpatient settings or case registers covering a circumscribed geographical area, such as the catchment area of a hospital, while relatively less is known about the general population [9,14,15]. For example, a retrospective Canadian cohort study, using administrative healthcare data of 19,041 individuals with ED from 1990–2013, showed that individuals with EDs identified in hospital settings had roughly a five-fold higher mortality rate relative to the general population [16]. The age, sex, and place of residence-adjusted HR for all-cause mortality of EDs in a longitudinal study in Finland among 2450 adults referred to a tertiary care-level ED unit was 3.54 (95%CI 2.52–4.96) [14]. The mortality associated with different types of mental disorders, including EDs, schizophrenia, mood disorders, personality disorders, and behavioral disorders, was also investigated in a recent population-based cohort study in Denmark on 7,369,926 people (23,196 persons with ED) younger than 95 years of age from 1995–2015. Similar to the previous studies, diagnosed individuals in this study also only included those with mental disorders registered in psychiatric inpatient, outpatient, and emergency settings. Therefore, one of the limitations of this cohort was a vulnerability to selection bias arising from a lack of representation of patients who are only treated by a general practitioner or who do not seek specialized help for their mental health. The CMR per 1000 person-year for EDs in this study was 3.0; however, those for schizophrenia and mood disorders were higher (28.1 and 31.2, respectively) [17]. Nevertheless, these aforementioned findings on mortality of EDs may not be representative of a general population and may not be generalizable to a greater range of cases of EDs. They may represent a very small and distinct proportion of the wider EDs population [18,19,20]. Since it has been revealed that despite the elevated contact with health care services among people with EDs, somewhere between 67% and 83% of cases fail to engage with treatment after referral [21,22]. A wide range of factors lead to this unmet need for treatment, such as difficulty accessing specialist services, the financial cost of treatment, perceived shame, and stigma attached to EDs [19,21]. Therefore, an important question to be answered is whether EDs are associated with higher mortality in the general population.

For epidemiological surveys of EDs in the general population, a self-reported current and lifetime screening tool using single-item questions, such as “Have you ever had anorexia”, has been shown to have a reliable specificity and sensitivity [23]. Moreover, the validity of self-reported health in population-based studies has repeatedly been confirmed and even found to be a stronger predictor of associated mortality than instruments explicitly designed for this purpose [24]. However, to our knowledge, there is no empirical research available to assess the mortality of EDs based on the self-reported current and lifetime history of EDs in the general population. Nevertheless, these findings will assist health care providers and policymakers in public health messages about the serious consequences of eating disorders. While this study was conducted in Canada, the results are likely to apply to the general populations of other developed countries. However, to the extent that cultural, health system, or other between-country differences may affect the association of EDs with mortality, the results are most directly supportive of a need to address the issue of mortality in people with EDs in Canada. Hence, the main objective of the current study was to investigate the mortality of self-reported lifetime history of EDs among participants in the population-representative Canadian Community Health Survey linked to national mortality data, which covers all of the general Canadian population.

## 2. Materials and Methods

### 2.1. Data Source

**Figure 1:**
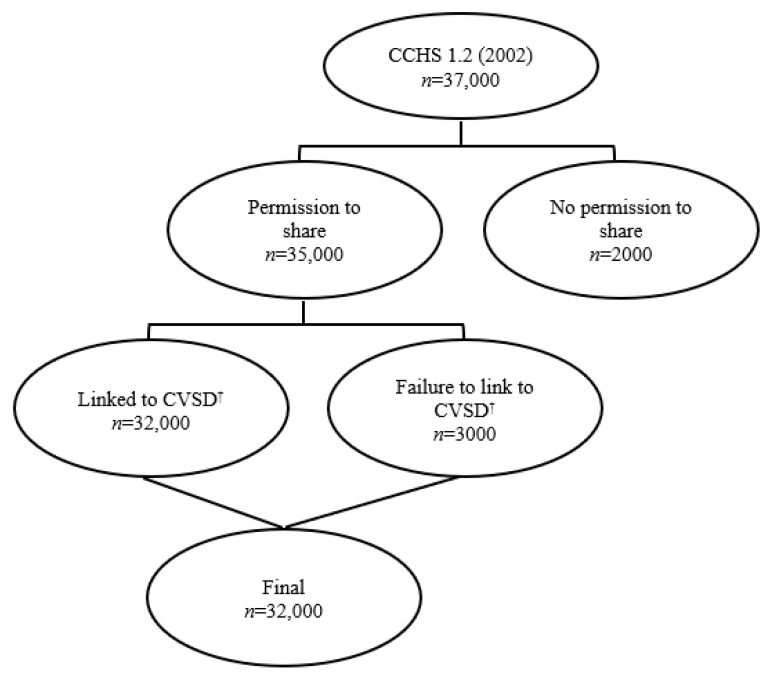
Flow diagrams for data linkage (estimates are rounded in keeping with Statistics Canada data release guidelines). ^†^ CVSD, Canadian Vital Statistics Database.

The data source was the Canadian Community Health Survey (CCHS) 1.2, also known as the CCHS mental health and well-being survey conducted by Statistics Canada [25]. The CCHS is a cross-sectional survey that collects information related to mental health status, mental health care utilization, and mental health determinants for the Canadian population. This survey was conducted in 2002 (between May and December), and the sample size was 36,984 with a 77% response rate (Figure 1) [26]. The sampling method was a multi-stage stratified cluster design. The inclusion criteria in this national survey consisted of noninstitutionalized people aged 15 years or older living in private dwellings in the 10 Canadian provinces [26,27]. The exclusion criteria contained individuals residing in the three territories, on reserves and other Aboriginal settlements in the provinces, the clientele of institutions, children aged 15–17 that are living in foster care, the full-time members of the Canadian Forces, and residents of some remote areas, groups that, in total, exclude less than 3% of the general population [26,28]. A “share file” that included the participants who provided consent for their data to be linked to other data sources was subsequently linked, by Statistics Canada, to the Canadian Vital Statistics Database (CVSD), allowing confirmation of vital status and, where relevant, and date of death, see Figure 1. A detailed description of data linkage procedures and their quality assessment has been reported elsewhere [29,30]. The linked data are available to researchers through the Canadian Research Data Centres Network. The current analysis took place in the Prairie Regional Data Centre on the University of Calgary Campus [29,31]. 

### 2.2. Measures 

The demographic information of the entire population, including age, sex, employment status (currently working versus not employed), the highest educational level, and marital status in three groups: “Married/Common-law” (in Canada, common-law and legally married spouses have very similar legal standing [29]), “Single”, i.e., never married, and “Widow/Separated/Divorced” were collected using field-tested items. Diagnosis of the EDs, schizophrenia, and mood disorders such as depression, bipolar disorder, mania or dysthymia, and post-traumatic stress disorder (PTSD), were assessed in each survey’s “chronic conditions” module. The wording of the item was similar in each survey: “Remember, we’re interested in conditions diagnosed by a health professional”. For example: “Do you have an eating disorder such as anorexia or bulimia?”. The interview questions for these disorders were those of a Canadian adaptation of the World Mental Health version of the Composite International Diagnostic Interview (WMH-CIDI) [26]. The WMH-CIDI is a lay-administered psychiatric interview that generates a lifetime profile of a person with a disorder defined partly according to both the 10th version of the International Statistical Classification of Diseases and Related Health Problems (ICD-10) and the Diagnostic and Statistical Manual of Mental Disorders, 4th Edition (DSM-IV) [26]. 

BMI was calculated by dividing the self-reported participants’ weight in kilograms by the square of their self-reported height in meters (kg/m^2^). The low BMI for participants younger than 18 years old was defined as less than the tenth percentile for age and sex [32], and for older participants below 18.5 kg/m^2^, as recommended by the World Health Organization (WHO) criteria [33,34]. Binge drinking was assessed with a question in the CCHS survey “How often in the past 12 months have you had five or more drinks on one occasion?”. Binge drinking was defined if the response was “more than once a month”. Smoking to cope with stress was determined based on the question “When dealing with stress, how often do you try to feel better by smoking more cigarettes than usual?” if the response was “sometimes” or more.

All of the variables mentioned above were assessed using field-tested survey modules developed by Statistics Canada, which were made available in multiple languages. Detailed information on each of the surveys, questionnaires, and user guides are available from the 2002 mental health survey documentation [25,29,35].

### 2.3. Data Analysis

The interview date for each participant is recorded in the survey, which is considered the baseline time point. The participants were classified as exposed or not exposed to EDs according to their answers in the survey. Time to death was calculated as the difference between the date of death and the interview date. In the linked data, both the date and underlying cause of death for those who subsequently died were recorded. Time to the event (death) was calculated by subtracting the date of the baseline interview from the date of death [29]. Those surviving up to the linkage date (31 December 2017) were censored at that date. Covariates were derived from the survey dataset and were, therefore, all recorded at baseline. No information on time-varying covariates was available. 

The stratified multi-stage sampling procedure used in CCHS leads to unequal selection probabilities. Therefore, the estimates should be analyzed using a set of 500 replicate bootstrap-sampling weights to ensure population representativeness. A procedure for bootstrap-weighting recommended by Statistics Canada was used in this analysis [28].

As in most studies of mortality, a time-to-event method of analysis was considered most appropriate due to its ability to address differing person time contributions by study participants. Therefore, after confirming the proportional hazard assumption, a Cox proportional hazards model was used to estimate hazard ratios from the time to event data [36] using Stata version 16. An unadjusted HR (crude HR) was estimated using a model that contained EDs but not covariates. While the CCHS dataset contains a rich set of covariates, the number of participants with EDs who died was too small to produce models with multiple covariate adjustments; therefore, covariates in this study included age (treated as a continuous variable) and sex. Confounding was observed only by age; however, to be consistent with other EDs mortality studies, the impact of sex and both age and sex were also assessed by adding each variable (along with an interaction term) one at a time to the model. For all analyses, the alpha level was set at 0.05.

## 3. Results

This study sample was restricted to respondents age 15 years and older at the time of the survey (consisting of approximately 31,130 respondents and mean age = 43.95 years). As shown in Table 1, 0.5% reported a positive lifetime history of EDs. About 14.4% of these individuals died, 89% were female, 52.5% were single, most had a post-secondary graduate degree (64.4%), and 11.5% reported a low BMI. 

A Cox proportional hazard model showed that the unadjusted HR (crude HR) for the lifetime history of an ED was 1.35 (95% CI 0.70–2.58). When the model was adjusted for age and then both age and sex, the HR increased to 4.4 (95% CI 2.2–8.0) (data not shown) and 4.54 (95% CI 2.33–8.84, Table 2), respectively.

For purposes of comparison, we generated unadjusted and age/sex-adjusted HR for other self-reported history of mental health disorders and low BMI in the same population. As shown in Table 2, the unadjusted HR for schizophrenia/psychosis (1.44, 95% CI 1.04–1.99) was higher than the crude HR for ED, however, the age/sex-adjusted HR (2.02, 95% CI 1.24–3.31) was smaller than the age/sex-adjusted HR for EDs. Similarly, the age/sex-adjusted HR for the self-reported history of mood disorder and PTSD was lower than that of eating disorders. For comparison, age/sex-adjusted HRs for other potential determinants of mortality are also presented in Table 2: binge drinking, smoking to cope with stress (which is here considered a proxy for smoking), and low BMI.

## 4. Discussion

To our knowledge, this is the first study that evaluated all-cause mortality of self-reported lifetime history of all EDs in a general population. This study confirms the high mortality in individuals with a lifetime history of ED that is not due to the selection of severely ill respondents as in prior studies of clinical individuals. However, as the prevalence of EDs was low, it is likely that these general population estimates also reflect a subset of the entire population with EDs. Previous studies reported mortality in a broad category of EDs [8,16,17] or with DSM or ICD diagnosis of anorexia nervosa [37,38], bulimia nervosa [37,38], other specified feeding or eating disorders [37], or binge eating disorder [14] based on a selective population, such as health-administrative data, inpatients-settings, or outpatients care. Therefore, the generalization of these results to all patients with EDs was limited only to those in treatment settings [14]. The current study helps to confirm that mortality is substantially elevated in members of the general population who report that they have been diagnosed with an ED, including those with subclinical eating disorders or those who failed to engage with treatment once referred. There is widespread stigma toward people affected by EDs by the general public, medical professionals, and service users due to the attribution of personal responsibility for illness behaviors [39]. Therefore, the seriousness of EDs may be underreported as a result of individuals who trivialize and minimize associated psychological and medical challenges [40]. There is also a self-stigma in people with EDs that leads them to deny, minimize, or assign positive meaning to their behaviors [39,41]. The effect of public/self-stigma may lead people to be reluctant to seek treatment, leave treatment prematurely, or experience a loss in the necessary components of recovery [42]. The current findings could be integrated into psychoeducation material for adolescents/adults with ED and their caregivers to help challenge the view that these illnesses are not serious, necessitating treatment. Public health campaigns could further highlight the high mortality rate of EDs [43].

Evidence has shown that mortality in EDs is influenced by age, sex, and case severity [6,9,16,44]. Generally, older individuals have an elevated risk of mortality for all types of EDs compared to the mortality of younger individuals [8], probably reflecting the strong effect of age and chronicity of illness on mortality. Consistent with this, age-adjusted estimates show a strong association with mortality. In a dataset of English national Hospital Episode Statistics, the SMR for the 15–24 age group diagnosed with anorexia nervosa or bulimia nervosa was found to be 11.5 and 4.1, respectively [8]. The SMR for the 25–44 age group diagnosed with anorexia nervosa or bulimia nervosa was found to be 14.0 and 7.7, respectively [8]. A number of studies have also reported sex differences in ED mortality [44,45,46]. Although the lifetime prevalence of EDs among males is lower than females, the CMR and SMR observed in males are almost two-fold higher than in females [16,17]. These provide evidence for the potential impact of age and sex as covariates in the all-cause EDs mortality assessment. Therefore, in the current study, the HR for eating disorders was reported as adjusted for age, sex, and both age and sex. 

A naïve interpretation of crude HRs suggests only a weak effect of EDs (1.35, 95% CI 0.70–2.58, since the low end of the interval is below one); however, the age/sex-adjusted HR for EDs is much higher than that of other major mental health problems. This indicates that the unadjusted estimates were strongly confounded by age and sex, information that will be useful in planning the analysis of future studies. The direction of the confounding is predictable, since EDs were reported more often by younger people and by women (see Table 1), both of whom have lower rates of mortality. 

In this study, only for the purpose of comparison, the mortality associated with some self-reported lifetime major mental health problems, and low BMI calculated based on self-reported height and weight were also evaluated. Evidence shows that low BMI associated with EDs may affect mortality [4,7,47]. About 12% of the individuals with a self-reported lifetime history of ED and 2% of the entire population reported a low BMI. However, the causes of this low BMI are not distinguishable between those who were initially underweight and those who became underweight due to malnutrition, over-exercising, or other comorbidities [48]. The present study aimed to evaluate all-cause mortality of self-reported history of EDs; however, numerous previous studies have reported a different degree of malnutrition in patients with EDs [49,50,51]. Several lines of evidence on the effect of low BMI caused by malnutrition on mortality meets the key Bradford Hill’s criteria to establish this causal relationship [52,53,54,55]. 

In the current study, the age/sex-adjusted HR for lifetime history of EDs was over two-fold higher than the age/sex-adjusted HR for self-reported lifetime schizophrenia/psychosis. In this study, the self-reported lifetime schizophrenia/psychosis was based on the question asking whether they have schizophrenia or any other psychosis as diagnosed by a health professional (response options, yes or no). A previous study on the CCHS1.2 survey has shown that the prevalence estimates of these two self-report survey items provide what appears to be a plausible epidemiologic pattern [56]. Accordingly, the mortality associated with these two items might also follow the same pattern. A nationally representative cohort study in the UK using primary care electronic health records on over 11 million people reported a very similar adjusted HR (accounting for age, gender, calendar year, area-level deprivation, ethnicity, and the average number of visits to the physician per year of follow-up) for schizophrenia (2.08, 95% CI 1.98–2.19) to our study [57]. In the current study, the results in the general population are in line with the previous studies on the clinical samples that observed lower crude HR and higher sex/age-adjusted HR in EDs than schizophrenia [8,17,58,59]. 

Although the sex ratio of the participants in this study was 1:1, males reported a history of lifetime ED nearly eight times less than females (11.86% vs. 88.98%). This difference is much larger than the existing literature on the lifetime prevalence of EDs among different sexes measured with a standardized tool (2.2% vs. 8.4%) [1]. This discrepancy may be associated with the difference between females and males in diagnosis, self-identifying, and self-reporting lifetime EDs due to the immense stigmatization toward males with EDs, stereotypes linked to EDs, and a misdiagnosis by a specialist treatment center [60,61,62]. These current findings may indicate the importance of gendered issues in diagnosing and treating patients with EDs and the necessity of tailored services to the patient’s need, such as the same-sex therapeutic groups [61]. 

In the current study, mood disorders were not associated with mortality, which is surprising since CCHS respondents (linking data from four surveys: CCHS 1.1 (2000/2001), CCHS 1.2 (2002), CCHS 2.1 (2003/2004), and CCHS 3.1 (2005/2006)) with symptoms of major depressive episodes (according to versions of Composite International Diagnostic Interview, a structured interview that does not depend on help-seeking) do have elevated all-cause mortality [29]. As the variable included in this analysis was self-reported diagnoses of mood disorders, it is possible that help-seeking and potentially treated individuals with major depression do not have elevated mortality, in contrast to what was reported here for EDs.

### Limitations

In this study, not all the potential confounding effects in the analysis were considered. Relevant variables include the age of onset and duration of EDs, specific type of ED, information on treatment, dietary intake, suicidal ideation/attempts, and potential comorbidities. Prior studies on ED patients suggested that these factors predicted the time to death [4,63]. According to Statistics Canada regulations, the number of these variables in the current study was insufficient to release the data (such as suicidal ideation/attempts) or were not measured in the data source. However, the current study was not designed to make a causal statement of the effects of EDs, independent of other variables, on mortality, but rather to describe the pattern of mortality in this population. 

Another limitation is the lack of information regarding if the EDs cases also include binge eating disorder and other specified feeding or eating disorders, since in this study, the screening of current and lifetime EDs was based on a dichotomous question “Do you have an eating disorder such as anorexia or bulimia?”. While the self-reported current and lifetime-screening questions have a reliable specificity and sensitivity, bulimia nervosa compared to other ED types has poorer reliability in identifying true cases [23], which might be one of the reasons that the prevalence of EDs in our population is lower than other reports based on psychometric questionnaires. However, another possible reason for this prevalence discrepancy might be because they represent more severe groups, i.e., those who have sought professional help [23]. 

CCHS in Canada represents over 97% of the Canadian population aged ≥15, which covers the ten provinces and the three territories, while excluding those persons who meet the exclusion criteria [25,26]. Therefore, these findings cannot be generalized to these groups. Furthermore, in Canada, health care is based on a universal healthcare system, meaning that it is largely based on need rather than the ability to pay, permitting Canadians to have access to most healthcare services [64]. A universal healthcare system reduces, though does not eliminate, the potential inequalities in accessing care for the initial diagnosis of EDs by a healthcare provider among people with different socioeconomic backgrounds [64,65].

## 5. Conclusions

To our knowledge, the current study, for the first time, has reported the all-cause mortality of self-reported lifetime history of EDs in a population-based study. This finding highlights the seriousness of these disorders and supports the idea that there is an urgent need for strategies that may help to improve long-term outcomes, such as a need for public education for early diagnosis and a long-term plan for adequate early intervention. Different types of research would be needed to understand the underlying causal mechanism and, consequently, formulate a preventive strategy.

## Figures and Tables

**Table 1:** Characteristics of the population ^†^.

		Entire Population (95% CI)	History of Eating Disorder (95% CI)
History of an Eating Disorder (%)		0.47 (0.47–0.47)	-
Mean Age (Year)		43.95	32.96
All-Cause Death (%)		12.9 (12.9–13)	14.4 (14.2–14.6)
Sex (%)	Male	49.15 (49.13–49.17)	11.86 (11.68–12.05)
	Female	50.85 (50.83–50.87)	88.98 (88.80–89.16)
Marital Status (%)	Married/Common-law	62.04 (62.02–62.06)	29.66 (29.40–29.92)
	Single	25.11 (25.09–25.12)	52.54 (52.26–52.83)
	Widow/Separated/Divorced	12.85 (12.84–12.87)	17.8 (17.58–18.02)
Employment Status in the Past Week (%)	Working	63.84 (63.82–63.86)	44.44 (44.16–44.73)
	Absent	4.73 (4.72–4.74)	6.83 (6.69–6.98)
	No job/Permanently Unable to Work	31.44 (31.42–31.46)	47.86 (47.58–48.15)
Education (%)	<Secondary Graduate	11.54 (11.53–11.56)	15.25 (15.05–15.46)
	Secondary Graduate	13.15 (13.13–13.16)	11.02 (10.83–11.20)
	Some Post-Secondary	7.08 (7.07–7.09)	10.17 (10.00–10.34)
	Post-Secondary Graduate	68.23 (68.21–68.25)	64.41 (64.13–64.68)
Low BMI (%)		1.98 (1.98–1.99)	11.54 (11.32–11.76)

^†^ CI—Confidence Interval, BMI—Body Mass Index (definition of low BMI: for ages <18, below the 10th percentile for age and sex, and for ages >18, below 18.5 kg/m^2^).

**Table 2:** Crude and age/sex-adjusted effect of the self-reported major mental health problems on mortality ^†^.

	Crude HR (95% CI)	Age/Sex-Adjusted HR (95% CI)
Eating Disorder	1.35 (0.70–2.58)	4.54 * (2.33–8.84)
Schizophrenia/Psychosis	1.44 * (1.04–1.99)	2.02 * (1.24–3.31)
Mood disorder	0.60 (0.26–1.39)	1.07 (0.49–2.35)
PTSD	1.11 (0.80–1.55)	1.45 * (1.03–2.03)
Binge Drinking	0.55 (0.49–0.63)	1.39 * (1.20–1.62)
Smoking to Cope with Stress	0.91 (0.83–0.99)	1.82 * (1.65–2.00)
Low BMI	1.92 * (1.27–2.90)	2.97 * (1.93–4.59)

^†^ CI—Confidence Interval, HR—Hazard Ratio, PTSD—Post-Traumatic Stress Disorder, BMI—Body Mass Index (definition of low BMI: for ages < 18, below the 10th percentile for age and sex, and for ages >18, below 18.5 kg/m^2^). * Indicates statistical significance at the *p* < 0.05 level.

## Data Availability

The data used in this analysis are available through the Research Data Centers Program. Information is available at: http://www.statcan.gc.ca/eng/rdc/index (accessed on 21 September 2021).
